# Progressive striatal necrosis associated with anti-NMDA receptor antibodies

**DOI:** 10.1186/1471-2377-13-55

**Published:** 2013-05-31

**Authors:** Charalampos Tzoulis, Christian Vedeler, Mette Haugen, Anette Storstein, Gia Tuong Tran, Ivar Otto Gjerde, Martin Biermann, Thomas Schwarzlmüller, Laurence A Bindoff

**Affiliations:** 1 Department of Neurology, Haukeland University Hospital, Bergen 5021, Norway; 2 Department of Clinical Medicine, University of Bergen, Bergen, Norway; 3 Center for Nuclear Medicine/PET, Department of Radiology, Haukeland University Hospital, Bergen, Norway; 4 Institute for Surgical Sciences, University of Bergen, Bergen, Norway

**Keywords:** Dystonia, NMDA, MRI, PET, Encephalitis

## Abstract

**Background:**

We report a case of childhood onset, generalized dystonia due to slowly progressive bilateral striatal necrosis associated with anti-N-methyl-D-aspartate receptor (NMDAR) antibodies. This clinical phenotype has not been previously associated with NMDA receptor autoimmunity.

**Case presentation:**

An eighteen year old man presented with a history of childhood-onset, progressive generalized dystonia. Clinical examination revealed a pure generalized dystonia with no cognitive or other neurological findings. Magnetic resonance imaging showed bilateral high T2 signal striatal lesions, which were slowly progressive over a period of nine years. New parts of the lesion showed restricted water diffusion suggesting cytotoxic oedema. Positron emission tomography of the brain showed frontal hypermetabolism and cerebellar hypometabolism. Antibodies against the NR1 subunit of the NMDA receptor were detected in the patient’s serum and cerebrospinal fluid. There was no neoplasia or preceding infection or vaccination.

**Conclusion:**

This is the first report of chronic progressive bilateral striatal necrosis associated with anti-NMDAR antibodies. Our findings expand the clinical spectrum of disease associated with anti-NMDAR antibodies and suggest that these should be included in the work-up of dystonia with striatal necrosis.

## Background

Generalized dystonia due to bilateral striatal necrosis (BSN) has a heterogeneous etiology including hereditary, toxic, infectious and immune mediated disorders. Immune mediated BSN is a rare clinical entity [1] which has been associated with a variety of infectious agents including streptococci [2] and mycoplasma [3]. An infectious or parainfectious pathomechanism has been proposed, but the pathogenesis of these disorders remains unclear.

Basal ganglia involvement occurs commonly in the encephalitis associated with anti-N-methyl-D-aspartate receptor (NMDAR) antibodies. This acute or subacute illness can manifest psychiatric and neurological symptoms including epileptic seizures, autonomic instability, hypoventilation and decreased levels of consciousness. Patients often develop hyperkinetic movement disorders including choreoathetosis, myoclonus and dystonia [4-7] during the course of the illness, but chronic progressive dystonia has not been reported. While magnetic resonance imaging (MRI) of the brain may show T2 hyperintense lesions in various parts of the brain including the basal ganglia, progressive BSN has never previously been described [5-8]. Diagnosis is confirmed by detecting autoantibodies against the NR1 subunit of NMDA-type glutamate receptors in serum or CSF [5,7]. NMDAR encephalitis may be associated with neoplasms, usually teratomas of the ovaries or testes, but may also be postinfectious or idiopathic. Treatment consists of tumor resection and administration of immunosuppressant therapy usually in the form of intravenous immunoglobulin (IVIg) or plasma exchange with substantial recovery in about 75% of the cases [6,8].

We report a case of childhood onset, pure generalized dystonia due to slowly progressive BSN, associated with anti-NMDAR antibodies. Our patient’s presentation, course and clinical features have not been previously associated with anti-NMDAR autoimmunity.

## Case presentation

The patient, now an eighteen year old man, was born after normal pregnancy and delivery and had normal early psychomotor development. From the age of three years he experienced episodic migraine-like headache, which resolved spontaneously at the age of sixteen. From the age of nine he noticed slowly progressive difficulties with handwriting due to focal stiffness and abnormal postures of his right hand. There was no infection or vaccination prior to the onset of symptoms. Subsequently, he developed abnormal postures and involuntary movements in his whole right upper limb described as slow, strained abduction and elevation of the shoulder and flexion of the elbow, wrist and fingers. The motor symptoms gradually progressed and, over a period of approximately one year, involved both upper limbs, the neck and face, truncal muscles and the lower limbs. From the age of twelve years he lost unsupported locomotion. He is now eighteen years old and wheel-chair dependent with severe generalized dystonia. He has completed his primary and secondary education and shows no evidence of cognitive or psychiatric dysfunction.

Physical examination by the authors at the age of fifteen revealed severe generalized dystonia with predominantly tonic features, axial and appendicular rigidity and abnormal posturing involving the neck, trunk and all four extremities (Additional file 1). He had facial hypomimia and dystonia, and oromandibular dystonia with severe dysarthria, but no dysphagia or dysphonia. Sensory and cerebellar functions were normal and his cognitive function was unremarkable.

**Figure 1:**
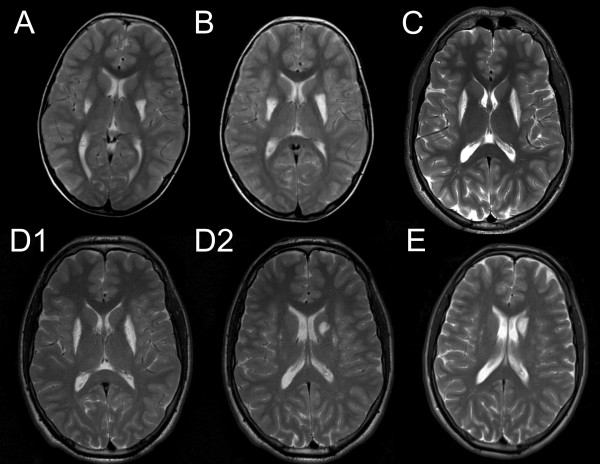
Axial T2-weighted MRI showing progression of the striatal lesions at the ages of nine (A), ten (B), fourteen (C), seventeen (D1&D2) and eighteen (E) years.

**Figure 2:**
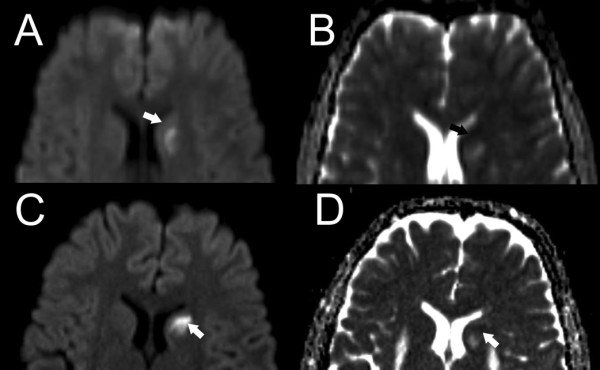
**Axial diffusion weighted scans (b1000) and corresponding ADC maps showing the evolution of diffusion changes in the left caudate head at seventeen (A&B) and eighteen (C&D) years of age.** The newest parts of the lesion (arrow) show low ADC consistent with restricted water diffusion.

MRI of the brain at the age of nine showed bilateral, high T2 signal lesions of the posterior putamen, which were more pronounced on the left side. Subsequent examinations at the ages of ten, fourteen, seventeen and eighteen years showed progression of the lesions in a posteroanterior fashion gradually involving all of the putamen and extending into the left caudate nucleus (Figure 1). The putaminal lesions appeared cavitated on T1 and FLAIR sequences and showed high apparent diffusion coefficient (ADC) on diffusion weighted imaging (DWI). The left caudate lesion appeared oedematous with high T2 signal and heterogeneous water diffusion with low ADC corresponding to the newest parts of the lesion (Figure 2). The nucleus accumbens and pallidum were spared and no other abnormalities were seen in the brain. There was no contrast enhancement on repeated examinations.

**Figure 3:**
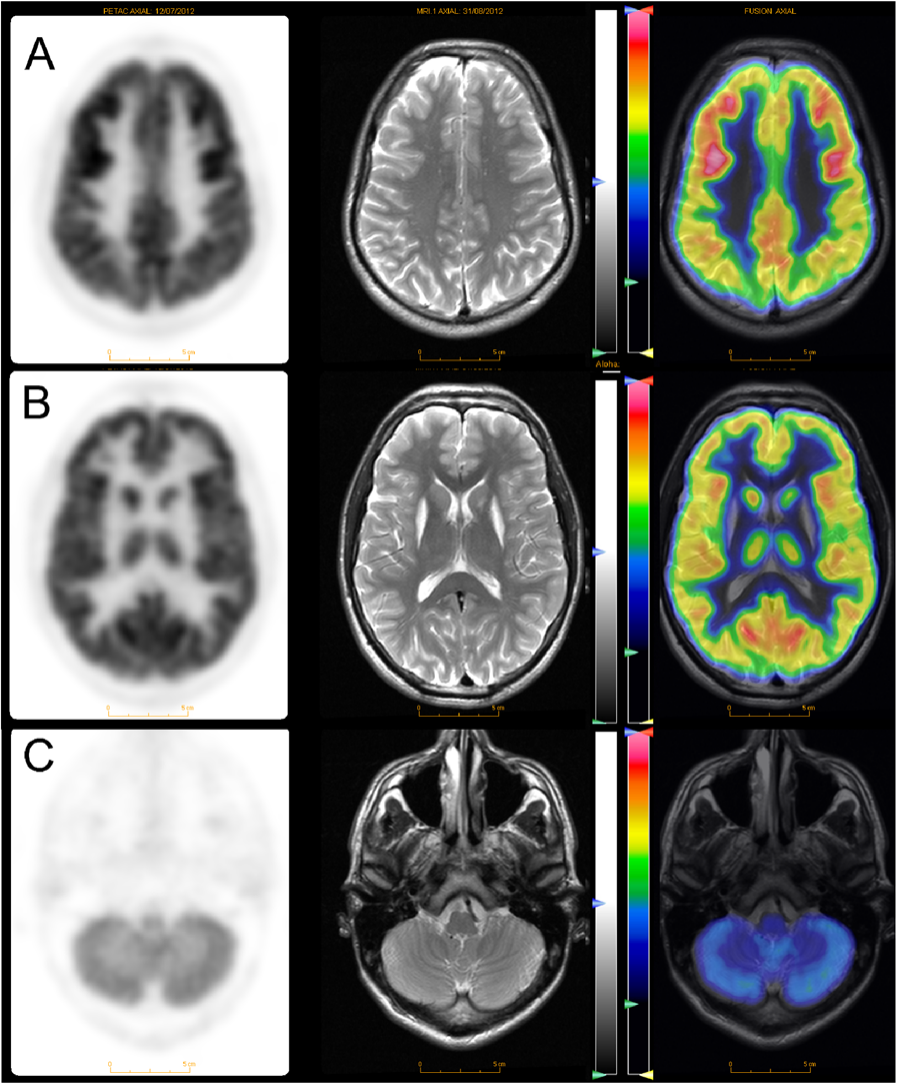
FDG-PET (left), axial T2 MRI (middle) and superimposed PET-MRI images of the brain showing frontal hypermetabolism (A), no detectable uptake in the putamina and reduced uptake in the left caudate (B) and hypometabolism in the cerebellum (C).

Whole body positron emission tomography (PET) imaging with fluor-18-deoxy-glucose (FDG) was performed at the age of eighteen on a Siemens Biograph 40 PET-CT. FDG-PET of the brain showed glucose hypermetabolism in the frontal cortex and hypometabolism in the cerebellum. There was no tracer uptake in the putamen and reduced uptake in the left caudate (Figure 3). PET-CT of the torso showed no abnormalities and ultrasound of the testes revealed no signs of teratomas or other neoplasms.

CSF analysis on several occasions showed normal cell count and mildly elevated protein (0.52-0.53 g/L), but no oligoclonal bands. Antibodies against the NR1 subunit of the NMDA receptor were detected using transfected cells produced by Euroimmun Medizinische Labordiagnostika AG (D-2356 Lübeck) in the patient’s serum and CSF. Screening for other autoantibodies to nervous system antigens was negative including anti-Hu, Ri, Yo, Tr, MAG, myelin, Ma/Ta, GAD, amphiphysin, AMPA, GABA-b receptor, LGI1, CASPR2 and glycine receptors.

He had normal metabolic screening in blood, CSF and urine. Wilson’s and Leigh disease due to mitochondrial DNA or *SURF1* mutations were excluded. He was thoroughly evaluated for mitochondrial disease including a muscle biopsy and qualitative/quantitative mitochondrial DNA analysis with normal findings. Analysis of *DYT1*, *RRM2B*, *SUCLA2* and *OPA1* genes was normal.

The patient received five intravenous immunoglobulin (IVIg) infusions (Kiovig 0.4 g/day) followed by two more infusions a month later and a new screening for anti-NMDAR antibodies one month later (2 months after treatment start) was negative in serum and CSF. Three months after the first IVIg infusion his clinical status and MRI findings, including lesional ADC, are stable but there have not been signs of clinical improvement. He receives regular intramuscular injections of Incobotulinum toxin A in the upper limbs resulting in moderate improvement in motor function.

This work has been deemed quality control by our local ethical committee (Regional Committee for Medical Research Ethics in Western Norway).

## Conclusions

We report a case of generalized dystonia due to BSN associated with anti-NMDAR antibodies. Unlike the cases of anti-NMDAR encephalitis, our patient has a pure, predominantly tonic dystonia with an insidious onset and slowly progressive course. The selectivity of the brain lesions is striking. There is involvement of the dorsal striatum (putamen and caudate nucleus), which is predominantly involved in motor function and sparing of the ventral striatal structures (nucleus accumbens, olfactory bulb), which are mostly connected to the limbic system and involved in cognitive and emotional functions. This localization correlates well with the clinical features of our patient who had a pure movement disorder with no signs of psychiatric or cognitive dysfunction or impaired consciousness.

One patient with isolated hemidystonia and anti-NMDAR antibodies has been reported [4]. Unlike our case, that patient had an acute onset and normal MRI and responded well to treatment. It is however possible that early treatment in that case prevented the development of striatal lesions and generalized dystonia.

The finding of restricted diffusion suggests cytotoxic edema affecting neurons in the newest parts of the lesions. The etiology of this damage is unclear, but may reflect direct antibody-mediated damage and/or excitotoxicity. Neuronal hyperexcitability is reported with anti-NMDAR antibodies [9] and the lack of contrast leakage in the patient’s lesions, suggesting an intact local blood-brain barrier, and minor CSF findings, would speak against an aggressive intrathecal inflammation.

Treatment with IVIg decreased the patient’s antibody titer below detection levels, but has thus far not produced apparent clinical effects. This is not surprising given the extensive damage in the patient’s striatum and the short observation time. On the positive side, his disease has not shown further progression, but longer follow-up is required before the treatment response can be confidently assessed.

While our study is based on only one case, it is highly unlikely that the anti-NMDAR antibodies in our patient are an incidental finding. Anti-NMDAR antibodies are rare and false positive occurrence in both serum and CSF has not been reported. A pathogenic role for the antibodies in our patient is also supported by the FDG-PET findings of frontal hypermetabolism and cerebellar hypometabolism, which although not specific, are consistent with findings reported in patients with encephalitis [10]. As the NMDR antibodies were detected at a late stage of disease in our patient, it could also be argued that they represent a secondary phenomenon e.g. due to chronic neuronal damage leading to peripheral “leakage” and immune presentation of cerebral antigens. This however is also unlikely as we found no evidence of blood-brain barrier dysfunction and detected no other auto-antibodies to neuronal antigens. Irrespective of whether the NMDAR antibodies are a primary or secondary phenomenon, it appears highly likely to us that they contribute to the neuronal damage in the striatum of our patient.

Our findings raise the possibility anti-NMDAR autoimmunity may be an unrecognized cause of dystonia with BSN. While further cases are needed to elaborate the mechanisms involved, we feel that our study highlights the need to thoroughly investigate cases with BSN as early identification may provide the opportunity for better and more effective treatment. We suggest, therefore, that anti-NMDAR antibodies should be included in the work up of chronic movement disorders with MRI evidence of striatal necrosis.

### Consent

Written informed consent was obtained from the patient for publication of this case report and any accompanying images or videos. A copy of the written consent is available for review by the Editor of this journal.

### Ethics approval

This work has been deemed quality controlled by our local ethics committee (REK vest).

## Abbreviations

ADC: Apparent diffusion coefficient; BSN: Bilateral striatal necrosis; CSF: Cerebrospinal fluid; FDG: Fluor-18-deoxy-glucose; IVIg: Intravenous immunoglobulin; MRI: Magnetic resonance imaging; NMDA: N-methyl-D-aspartate; NMDAR: N-methyl-D-aspartate receptor; PET: Positron emission tomography.

## Competing interests

The authors declare that they have no competing interests.

## Authors’ contributions

CT, LB: clinical evaluation, acquisition, analysis and interpretation of data, drafting of the manuscript. MH, CV, GT, MB, TS: analysis and interpretation of data, critical revision of the manuscript. LAB, AS, IOG, CV: critical revision of the manuscript. All authors read and approved the final manuscript.

## Pre-publication history

The pre-publication history for this paper can be accessed here:

http://www.biomedcentral.com/1471-2377/13/55/prepub

## Supplementary Material

Additional file 1 Video of the patient at the age of eighteen. Click here for file
